# Enhancement of Exchange Bias and Perpendicular Magnetic Anisotropy in CoO/Co Multilayer Thin Films by Tuning the Alumina Template Nanohole Size

**DOI:** 10.3390/nano12152544

**Published:** 2022-07-24

**Authors:** Mohamed Salaheldeen, Ayman Nafady, Ahmed M. Abu-Dief, Rosario Díaz Crespo, María Paz Fernández-García, Juan Pedro Andrés, Ricardo López Antón, Jesús A. Blanco, Pablo Álvarez-Alonso

**Affiliations:** 1 Physics Department, Faculty of Science, Sohag University, Sohag 82524, Egypt; 2 Departamento de Física, Universidad de Oviedo, C/Federico García Lorca 18, 33007 Oviedo, Spain; charo@uniovi.es (R.D.C.); fernandezpaz@uniovi.es (M.P.F.-G.); jabr@uniovi.es (J.A.B.); 3 Departamento de Física Aplicada, EIG, Universidad del País Vasco, UPV/EHU, 20018 San Sebastián, Spain; 4 Chemistry Department, College of Science, King Saud University, Riyadh 11451, Saudi Arabia; anafady@ksu.edu.sa; 5 Chemistry Department, Faculty of Science, Sohag University, Sohag 82524, Egypt; amamohammed@taibahu.edu.sa; 6 Instituto Regional de Investigación Científica Aplicada (IRICA), Universidad de Castilla-La Mancha, 13071 Ciudad Real, Spain; juanpedro.andres@uclm.es (J.P.A.); ricardo.lopez@uclm.es (R.L.A.); 7 Departamento de Física Aplicada, Universidad de Castilla-La Mancha, 13071 Ciudad Real, Spain; 8 Instituto Universitario de Tecnología Industrial de Asturias, Universidad de Oviedo, 33203 Gijón, Spain

**Keywords:** nanostructured thin films, micromagnetic simulation, domain walls, perpendicular magnetic anisotropy, exchange bias

## Abstract

The interest in magnetic nanostructures exhibiting perpendicular magnetic anisotropy and exchange bias (EB) effect has increased in recent years owing to their applications in a new generation of spintronic devices that combine several functionalities. We present a nanofabrication process used to induce a significant out-of-plane component of the magnetic easy axis and EB. In this study, 30 nm thick CoO/Co multilayers were deposited on nanostructured alumina templates with a broad range of pore diameters, 34 nm ≤ *D*_p_ ≤ 96 nm, maintaining the hexagonal lattice parameter at 107 nm. Increase of the exchange bias field (*H*_EB_) and the coercivity (*H*_C_) (12 times and 27 times, respectively) was observed in the nanostructured films compared to the non-patterned film. The marked dependence of *H*_EB_ and *H*_C_ with antidot hole diameters pinpoints an in-plane to out-of-plane changeover of the magnetic anisotropy at a nanohole diameter of ∼75 nm. Micromagnetic simulation shows the existence of antiferromagnetic layers that generate an exceptional magnetic configuration around the holes, named as antivortex-state. This configuration induces extra high-energy superdomain walls for edge-to-edge distance >27 nm and high-energy stripe magnetic domains below 27 nm, which could play an important role in the change of the magnetic easy axis towards the perpendicular direction.

## 1. Introduction

Modern discoveries in materials science have clearly demonstrated that the scalability of the intrinsic physical properties of materials can lead to the development of new or improved technological applications. Recently, nanostructured magnetic materials exhibiting unique magnetic moment configuration have attracted a great deal of attention because of their outstanding utility in mobile communications, biomedical sensors, logic circuits, and high-density data storage devices [1,2,3,4,5,6]. In this respect, decreasing the dimension of the nanostructured thin films near to its critical length could lead to significant differences in the magnetic properties compared to non-patterned thin films [7]. This is clearly evident in the exchange bias (EB) effect, a magnetic effect that usually appears at the interface of the antiferromagnetic (AFM) and ferromagnetic (FM) phases related to the exchange coupling between them [8,9,10,11]. The EB phenomenon is characterized by an enhancement of the coercive field (*H*_C_) and a displacement of the hysteresis loop along the applied magnetic field after the samples have been field-cooled [8,12]. Due to its interfacial origin, the magnitude of this loop shifts and the so-called exchange bias field (*H*_EB_, defined as *H*_EB_ = $\frac{H_{C1}+H_{C2}}{2}$, in which *H*_C1_ and *H*_C2_ are the left and right coercive fields, respectively) is inversely proportional to the thickness of the FM layer [13]. In view of their peculiar properties, magnetic materials unveiling strong EB behavior have gained much interest owing to their widespread applications in sensors [14], spintronic devices [15,16], drug carriers [17], and magnetic read heads for magnetic information storage devices [18]. In addition, AFM/FM multilayer (ML) thin films with strong perpendicular magnetic anisotropy (PMA) have become a key factor in improving magnetic logic chips, spintronic devices, and random-access memory devices [19,20,21].

In this regard, AFM/FM CoO/Co MLs represent an excellent system for investigating the EB phenomenon [22,23], since cobalt is a widely used FM material in magnetic media recording, and CoO orders antiferromagnetically exhibiting high magnetocrystalline anisotropy and moderate Néel temperature (*T*_N_ = 290 K) [24,25,26]. However, fabricating CoO/Co MLs with large perpendicular magnetic anisotropy is still challenging. Conventional methods to obtain CoO/Co with PMA use multilayer structures of AFM/FM/non-magnetic metal or metal oxide interfaces [27,28,29], but these methods may affect some magnetic parameters of CoO/Co, such as magnetization saturation (*M*_S_) and damping coefficient [28,30]. An alternative approach that has already proven its success with other materials, such as Co/Permalloy [31,32], consists of using antidot nanostructured templates, which allows tailoring the physical properties of any host-patterned material through the variation of its geometric parameters, such as the hole size and the neighboring interdistance [33]. In particular, it has been reported that, for FM/AFM antidot thin films, the coercivity and EB field can be engineered by controlling the thickness of the FM layer, the diameter of the pores, and the density of the pores [9,34,35,36]. Nonetheless, the magnetic behavior of CoO/Co-MLs-based antidot nanostructured thin films has been investigated in only a few works, although the specimens have shown in-plane easy magnetization direction [37]; the case of CoO/[Co/Pd] MLs represents, however, a noteworthy exception presenting PMA—although the antidot structure results in an enhancement, not an induction of PMA [38]. Therefore, if highly effective PMA could be achieved in CoO/Co, this standard material would become interesting for perpendicular bit-patterned magnetic storage media applications and magnetic random-access memory with spin transfer torque magnetic random-access memories [19,39].

With this intention, we have explored a route to induce a PMA on CoO/Co ML by nanostructuration. We have used a low-cost technique consisting of depositing magnetic films onto nanoporous anodic alumina (NAA) templates, which provides self-assembling nanopores with reproducible two-dimensional hexagonal symmetry through well-defined control of geometrical parameters, such as lattice symmetry, edge-to-edge distance, pore length, and pore size [33]. Furthermore, an almost perfect periodicity of the hexagonal ordering (equivalent to that obtained by lithographic methods) can be achieved by adding a pre-patterning step before the anodization process of the alumina surface [33,40].

In this contribution, via precise control and manipulation of the geometrical parameters of CoO/Co antidot thin films, we examine the induced changes in magnetic properties such as magnetic anisotropy and EB field. By varying the antidot hole diameter, *D*, from 32 to 93 nm while keeping the lattice constant (*p* = 108 nm)—corresponding to a reduction in the network width, i.e., the edge-to-edge distance, *W*, from 76 to 13 nm—we disclose the conditions for the change of the magnetic anisotropy easy axis direction from in-plane (INP) to out-of-plane (OOP).

## 2. Materials and Methods

### 2.1. Synthesis Method

The synthesis of the CoO/Co ML antidot arrays is divided into two main steps: the fabrication of NAA substrates with a constant lattice parameter *p* = 108 nm and pore diameter varying between 34 ± 2 nm and 96 ± 3 nm, and subsequent deposition of magnetic layers on it.

**Synthesis of the templates.** NAA membranes (area of 1.5 × 1.5 cm^2^, thickness of 500 μm) were fabricated by two-step mild anodization of an aluminum foil (99.999% purity). To improve the surface smoothness of Al foils, an electropolishing process was performed in a mixture of H_3_PO_4_ and H_2_SO_4_, then they were washed at 50 V in a perchloric acid and ethanol solution (1:3 vol., 9 °C) for 10 min, as described elsewhere [41,42]. As the first anodization step produces disordered pores, a second anodization step was performed (5 h duration). A chemical etching process was carried out in 6 wt% orthophosphoric acid at 40 °C for etching times, *t*_etch_, between 25 and 75 min, to obtain NAA membranes with different pore sizes that varied in the range 34 nm ≤ *D*_p_ ≤ 96 nm, as reported in our previous investigation [31,43].

**Deposition process.** Nonepitaxial [CoO/Co] MLs were deposited on NAA templates by thermal evaporation (Edwards E306A, Cheshire, UK) in ultrahigh vacuum at pressures below 10^−5^ Pa [33,44,45,46]. Reference continuous ML of the same composition was deposited onto a 0.1 mm thick glass substrate at room temperature. A 5 nm thick Pd layer was deposited as a buffer layer. Next, 1.5 nm of Co (99.99% purity) was deposited and oxidized for 10 min in an oxygen atmosphere at a pressure of 3 × 10^2^ Pa as reported elsewhere [20]. Then, the oxide layer was shielded by 0.9 nm of Co. After 7 repetitions of [CoO/Co], a 1 nm thick Pd layer (99.99%) was deposited as a capping layer to avoid oxidation. The layer thickness was controlled during the deposition process using a calibrated setup of crystal quartz and was confirmed with X-ray reflectivity measurements as we shall see later.

### 2.2. Samples Characterization

The hole diameter and the lattice constant of the nanostructured sample were measured using a high-resolution scanning electron microscope, HR-SEM (JEOL-6610LV). High-resolution transmission electron microscopy, HR-TEM, images were acquired with a JEM JEOL 2100 microscope operating at 200 kV to determine the nanostructure and crystallographic order of the films. The continuous sample was manually milled, and the resulting powders were dispersed in ethanol by ultra-sonication and localized in a carbon grid. X-ray reflectivity (XRR) measurements were taken on a conventional θ-θ reflectometer (Bruker D8 Advance, Berlin, Germany) equipped with a Göbel mirror, a knife edge filter, and Cu Kα radiation. XRR measurement data were fitted using commercial LEPTOS7 software, V7.10.12 (Bruker). The magneto-optical properties of the antidot arrays and continuous [CoO/Co] MLs were analyzed using a magneto-optical Kerr Effect, MOKE magnetometer. MOKE measurements were performed at room temperature in the INP direction using transverse T-MOKE, and in the OOP direction using polar P-MOKE. Complementary magnetic measurements of [CoO/Co] ML antidot and continuous thin films were obtained by a vibrating sample magnetometer, VSM, with applied magnetic fields up to 20 kOe, measured in the temperature range from 60 K to 300 K and in both INP and OOP directions to the film plane. The obtained data were corrected for the magnetic signal from the substrates (a glass in the case of the reference sample and alumina membrane for the antidot sample) and the sample holder.

### 2.3. Micromagnetic Simulation

Micromagnetic simulation of [CoO/Co]_2_ MLs was performed using OOMMF software [47]. The simulations were carried out for samples with a size of 500 nm × 500 nm × 6 nm, discretized with the unit cell of 2 nm × 2 nm × 0.5 nm, so that, in the Z direction, each Co layer was discretized in 3 cells FM and each CoO layer was split into 2 outer FM layers in contact with adjacent Co layers and a central AFM layer. For simulation, the standard parameters of the Co material were used: 1.4 × 10^3^ emu/cm^3^ as saturation magnetization and 3 × 10^−6^ as exchange constant. An exchange constant of −1.5 × 10^−6^ which is half that of Co was used for the CoO layer to ensure that *T*_N_ is lower than Curie temperature, as normally seen in AFM oxides relative to their native FM metals [48]. At the FM/AFM interface, a ferromagnetic interaction with the same cobalt parameters was considered.

## 3. Results

**Figure 1:**
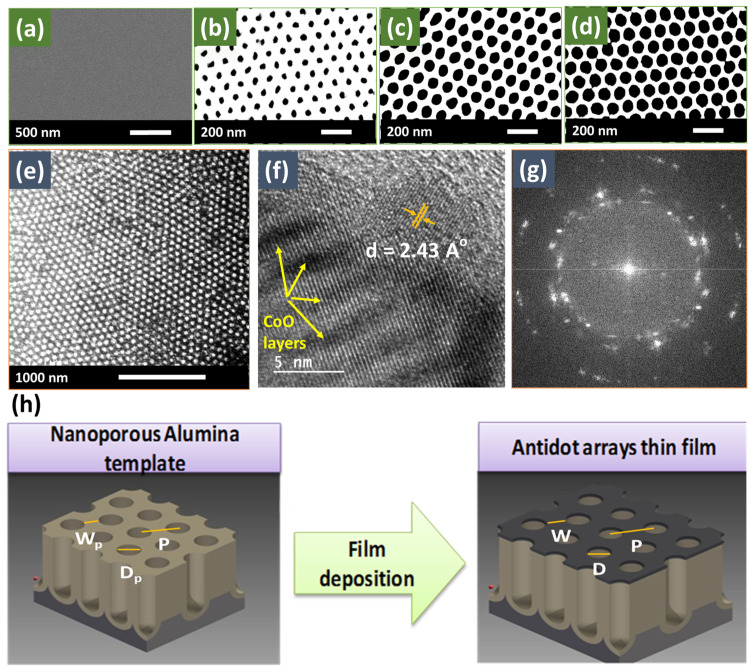
HR-SEM images of [CoO/Co] MLs deposited (**a**) on the top surface of a glass substrate to obtain the non-patterned sample as a reference, and (**b**–**d**) on the top surface of the NAA with different hole diameter. (**e**) HR-TEM image of [CoO/Co] ML antidot arrays taken at low magnification. (**f**) HR-TEM image of the continuous thin film obtained at high magnification, showing the interplanar distance of 2.43 Å corresponding to the (111) Bragg reflection of CoO; (**g**) shows the SAED pattern for continuous thin film, indicating coarse crystalline grains, as well as the first strongest Bragg diffraction rings (111) and (200) associated with the FCC crystal structure of both Co and CoO (see the text for further details); (**h**) illustrates the sketch of alumina membrane with its geometrical parameters before and after the MLs deposition.

All specimens were analyzed using HR-SEM to estimate the geometrical parameters of NAA templates and CoO/Co ML antidot array thin films before and after the deposition process, as summarized in Table 1. Well-ordered hexagonal arrangements of holes with *p* = 107 ± 3 nm were observed in all templates. Figure 1a illustrates the non-patterned CoO/Co ML sample with total thickness *t* = 30 nm, and Figure 1b–d plot three selected images of CoO/Co ML antidot samples (*S*_2_, *S*_4_, and *S*_5_, respectively) with different hole diameters. The lowest value of the magnetic surface coverage ratio, $C=\left(1-\frac{\pi D^{2}}{2\sqrt{3}p^{2}}\right)\times 100$ (see ref. [49] for further details), was obtained for CoO/Co ML antidot array samples with hole diameter 94 ± 3 nm (see Table 1), being lower than for other systems obtained using similar techniques [50,51,52]. The large variation of the magnetic surface coverage allowed us to investigate the influence of the geometric parameters of CoO/Co ML antidot films deposited on NAA on the exchange bias effect and the magnetic anisotropy. 

HR-TEM micrograph of a CoO/Co ML antidot array sample obtained after being released from the NAA is presented in Figure 1e, which shows that the thin film nanoholes successfully duplicate the structure of the highly hexagonal ordered NAA template. Figure 1f represents a high-magnification image of the non-patterned CoO/Co ML, in which the ML structure is visible. The existence of defined regions with clear periodicity of the atoms supports the notion that the film has a polycrystalline structure even though MLs have been deposited onto an amorphous SiO_2_ substrate, which has been previously observed in this system [24] and has been corroborated by means of selected area electron diffraction (SAED) (Figure 1g). All the observed rings detected can be indexed as Bragg reflections of face-centered cubic (FCC) crystal structures: rock salt of CoO [*a* = 4.25 (2) Å] and metallic cobalt [*a* = 3.57 (2) Å].

**Figure 2:**
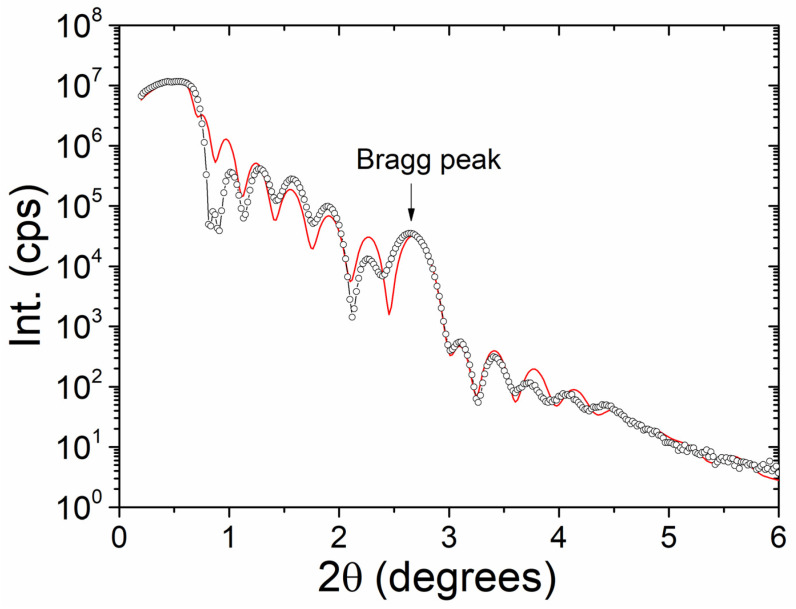
The X-ray reflectivity (XRR) data (open circles) and simulation (red line) of the non-patterned /Pd/[CoO/Co]_×7_/Pd ML thin films with layer thickness 30 nm.

The XRR measurement is plotted in Figure 2. We have fitted these data using the LEPTOS7 software, defining each layer by its thickness, density, and roughness. From the fit, we have determined the following thicknesses (in nm) for the continuous ML: Glass/Pd(4.0)/[CoO(1.9)/Co(1.4)]_×7_/Pd(0.9), values than agree fairly well with the nominal values—apart from the increase of the thickness of the oxidized Co layer. The estimated average roughness of the magnetic layers is about 0.8 nm, pointing out the formation of a moderately well-defined ML structure.

**Figure 3:**
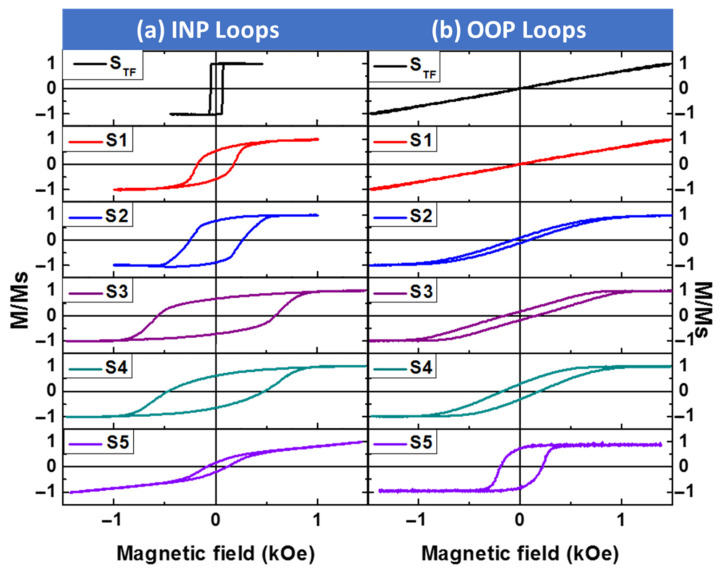
(**a**) INP and (**b**) OOP MOKE hysteresis loops for non-patterned and antidot CoO/Co ML samples with different hole diameters ranging from 34 ± 3 to 96 ± 3 nm and fixed layer thickness (*t* = 30 nm) and a constant lattice parameter (*p* = 108 nm) at room temperature (300 K).

**Figure 4:**
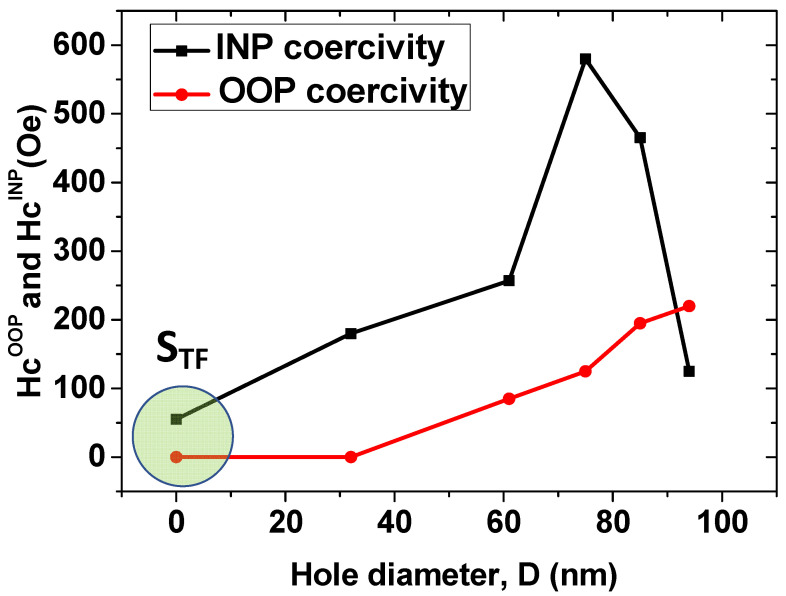
Room temperature coercivity measured along the OOP and INP directions for the antidot CoO/Co MLs with fixed layer thickness (*t* = 30 nm) and a constant lattice parameter (*p* = 108 nm) as a function of antidot hole diameter. Lines are guides for the eyes.

Room temperature surface magnetic properties of the non-patterned and antidot CoO/Co ML thin films have been measured along the INP and OOP directions by T-MOKE and P-MOKE, respectively. In Figure 3a,b, both the T-MOKE and P-MOKE hysteresis loops of the CoO/Co ML antidot films are represented; the results are plotted for patterned samples with nanohole diameters ranging between 32 and 94 nm, as well as for the continuous CoO/Co MLs of identical thickness, serving as a reference sample. Neither INP nor OOP hysteresis loops for any of the antidot or non-patterned samples exhibit an EB field at room temperature (300 K), which might be expected from the Néel temperature of CoO (*T*_N_ = 290 K) [30]. However, the magnetic anisotropy of the antidot array samples shows a dramatic change in its nature with the diameter of the antidot hole at room temperature: for continuous thin film (*S*_TF_) and nanopatterned specimens with *D* ≤ 75 nm (i.e., *S*_1_, *S*_2_, and *S*_3_), the magnetization is found to be along the INP direction, as a result of the high coercivity and remnant magnetization (relative to saturation magnetization) shown by the INP hysteresis loops. In fact, the maximum value of INP coercivity, *H*_C||_, of approximately 595 Oe was detected for the antidot specimen *S*_3_ with *D* = 75 nm, which is almost 12 times higher than the value of the coercive field obtained for the reference sample (see Figure 3 and Figure 4). Meanwhile, we found a huge saturating field and close to zero remnant magnetization along the OOP direction, as illustrated in Figure 3b. A significant decrease in *H*_C||_ (to 488 Oe) and an increase in the OOP coercivity, *H*_C__⊥_, (from 136 to 175 Oe) have been observed for the *S*_4_ antidot array sample, as plotted in Figure 4. Finally, a sharp reduction of *H*_C||_ has been detected for the *S*_5_ antidot sample accompanied by an increase in *H*_C__⊥_ (provoking *H*_C__⊥_ to become larger than *H*_C||_) and remanence in the OOP direction, as shown in Figure 3 and Figure 4, suggesting the existence of a dominant perpendicular component of the magnetic anisotropy.

The enhancement of the OOP magnetic anisotropy of CoO/Co MLs with the increase of antidot hole diameter is strongly related to the change in magnetostatic energy of the nanostructured system [33,53]. In addition, the increment of antidote hole diameter modifies the structure of the micromagnetic domain and creates different kinds of magnetic domain separated with superdomain walls with different energies, as reported elsewhere [11,51,54,55]. The growth of superdomain walls with high energy enhances, therefore, the OOP magnetic signal by increasing the antidote hole diameter, as illustrated in the micromagnetic simulation section (see below) and reported elsewhere [33,55].

**Figure 5:**
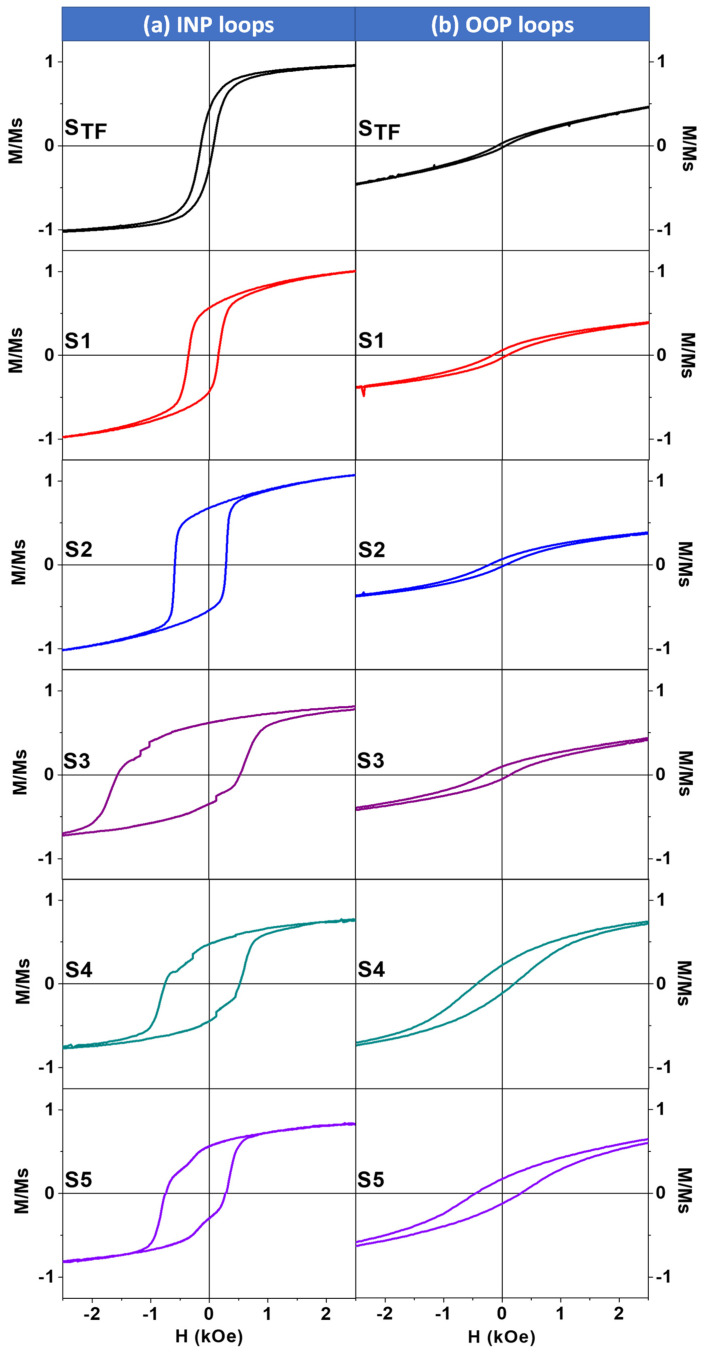
(**a**) INP and (**b**) OOP *M*(*H*) curves measured at 60 K after field cooling (*H*_cool_ = 20 kOe) for the CoO/Co MLs and (*S*_1_–*S*_5_) CoO/Co ML antidot array samples with different hole diameters ranging from 34 ± 3 to 96 ± 3 nm and fixed layer thickness (*t* = 30 nm) and a constant lattice parameter (*p* = 108 nm).

**Figure 6:**
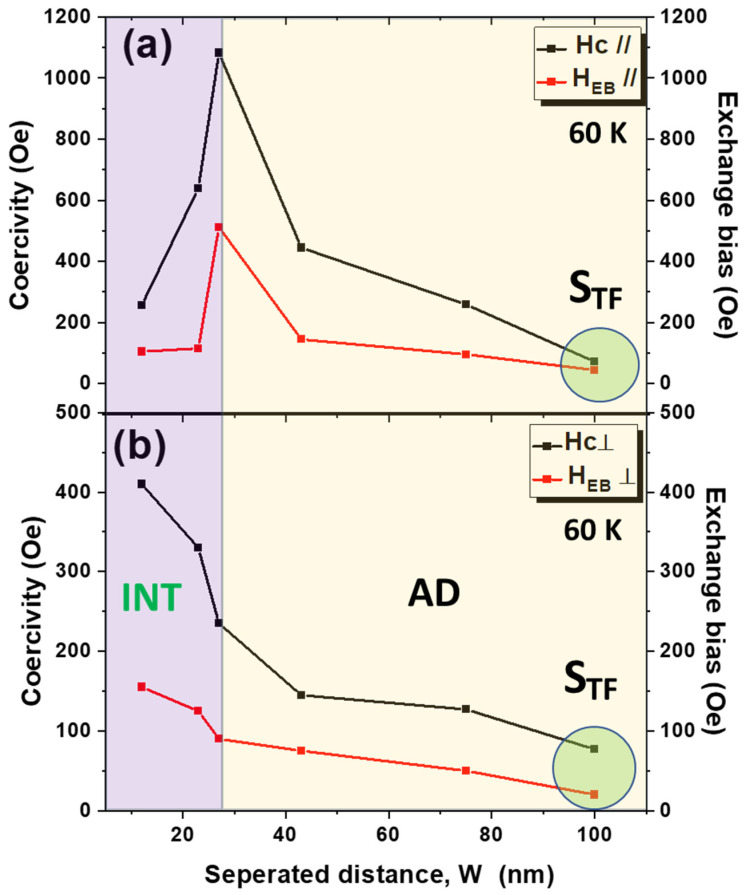
Variation of the coercivity and the EB field for continuous and antidot CoO/Co MLs with different edge-to-edge distance for (**a**) the in-plane and (**b**) the OOP direction at 60 K. Lines are guides for the eyes. We have assigned the value *W* = 100 nm to *S*_TF_ to facilitate comparison.

Figure 5 illustrates the INP and OOP hysteresis loops measured after field cooling under an applied magnetic field of 20 kOe (parallel to the film plane for INP loops and perpendicular to the film plane for OOP loops) from room temperature to 60 K for continuous and antidot CoO/Co ML samples. The variations of INP and OOP *H*_C_ and *H*_EB_ for the non-patterned and antidot array CoO/Co ML samples with different hole diameters at 60 K are plotted in Figure 6. The INP and OOP loops for the continuous film exhibit negative exchange bias fields of 45 and 20 Oe, respectively. For nanostructured samples, large comparisons to the reference thin film sample-coercivity and exchange bias fields in both directions are observed. The reader must note that, although CoO/Co system has shown high values of the EB, as reported by F. Radu et al. [56], the magnetic exchange bias field of CoO/Co system depends on several parameters, such as layer thickness, number of AFM/FM layers, temperature annealing, deposition conditions, and the method of the deposition, as discussed elsewhere [11,22,36,57]. *H*_C||_, *H*_C__⊥_, in-plane exchange bias field, *H*_EB||_, and out-of-plane exchange bias field, *H*_EB__⊥_, increase for the antidot array sample *S*_1_ to 259 Oe, 127 Oe, 96 Oe, and 55 Oe, respectively. By further enlarging the antidot hole, a monotonic increase in *H*_C__⊥_ and *H*_EB__⊥_ was observed with the antidot hole diameter. The maximums of *H*_C__⊥_ and *H*_EB__⊥_, reached for the *S*_5_ antidot array sample, are 410 Oe and 155 Oe, respectively (i.e., approximately 7 times higher than the corresponding values for the *S*_TF_ at the same temperature). In the case of *H*_C||_ and *H*_EB||_, both reach their maximums in the *S*_3_ antidot array sample, corresponding to 1.09 kOe and 0.55 kOe, respectively, although they are lower than the values reported for CoO/Co core/shell nanoparticles (*H*_C_ = 3.5 kOe and *H*_EB_ = 4.6 kOe) deposited without dilution in a paramagnetic matrix [58].

The reduction in *H*_C||_ observed for *S*_4_ and *S*_5_ antidot array samples agrees well with the trend noticed at room temperature, despite the observed reorientation of the magnetic anisotropy of *S*_5_ from the out-of-plane magnetic anisotropy at room temperature to the in-plane magnetic anisotropy at 60 K. Note also that INP loops for *S*_3_ and *S*_4_ show multistep magnetic behavior, which indicates a strong pinning effect induced by the antidot symmetry [59]. This multistep behavior visible in *M*(*H*) loops could be associated with edge defects at the nanostructure boundaries. Presumably, the hysteresis loops observed also show a signal originating from material that has entered the pores or was deposited on the edges and walls of the pores. In fact, this edge effect increases as the antidote hole diameter does. For this reason, the edge effect might play an important role in the reduction of in-plane coercivity and improvement of the OOP magnetic signal.

From the dependence of the INP and OOP *H*_C_ and *H*_EB_ on the values of *W* shown in Figure 6, we can distinguish two regimes:

1. Antidot (AD) regime: *W* > 27 nm. The antidot arrays are well disconnected from each other, and the expected tendency of both *H*_C||_ and *H*_EB||_ ∝ 1/*W* is detected, as illustrated in Figure 6a, as well as nucleation, hindering, and pinning of the magnetic domain walls. According to Castan-Guerrero et al. [54], this behavior is related to the mobility of the domain wall: when *W* decreases, there is less mobility of the domain wall, and *H*_C||_ and *H*_EB||_ increase.
2. Intermediate regime (INT): 0 < *W* ≤ 26 nm. When the distance between antidots is small enough, a modification in the *H*_C||_ and *H*_EB||_ behavior with the geometry is observed (see Figure 6a). In this case, as *W* is reduced (i.e., *D* increases), the propagation of the domain wall becomes harder and the linear dependence of *H*_C||_ and *H*_EB||_ on *W*^−1^ is gradually lost. With a further decrease of *W*, the edges of the holes become too narrow and the magnetic area that has to be nucleated with a reversed magnetic domain is reduced [54]. As a result, *H*_C||_ and *H*_EB||_ decrease.

## 4. Micromagnetic Simulations

For a better understanding of the magnetization reversal process of the CoO/Co AFM/FM nanopatterned thin films, we have performed micromagnetic simulation with OOMMF software for samples of Co/CoO/Co/CoO (the layers have been labeled as Co1, CoO1, Co2, and CoO2, respectively) nanostructured thin films with different hole diameters, and for non-patterned thin film as well for comparison. We have chosen four layers to reduce the time of simulation process while keeping reasonable accuracy that allows us to monitor the effect of adding AFM layers to FM layers. Moreover, we have concentrated on the magnetic structure at the remanence state to avoid any additional effect because of the external magnetic field. In addition, studying the remanence state of the samples gives us a clear description of their magnetization reversal process [54,55,60,61].

**Figure 7:**
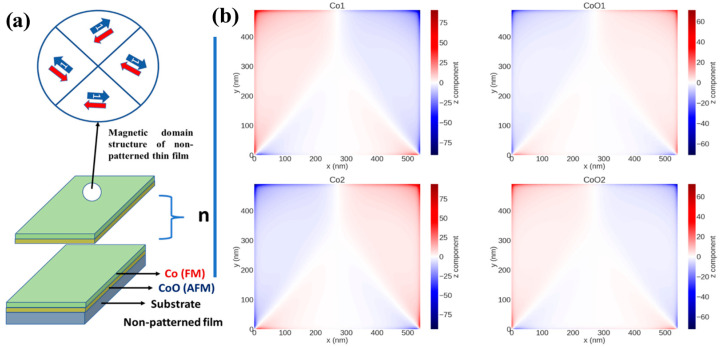
(**a**) Schematics of the spin configurations when the external magnetic field is parallel to the sample plane and sample layers distribution of CoO/Co non-patterned thin film. (**b**) Magnetic domain structure of four continuous layers of Co and CoO in the remanence state obtained by OOMMF when the external applied field was oriented along the x-axis.

**Figure 8:**
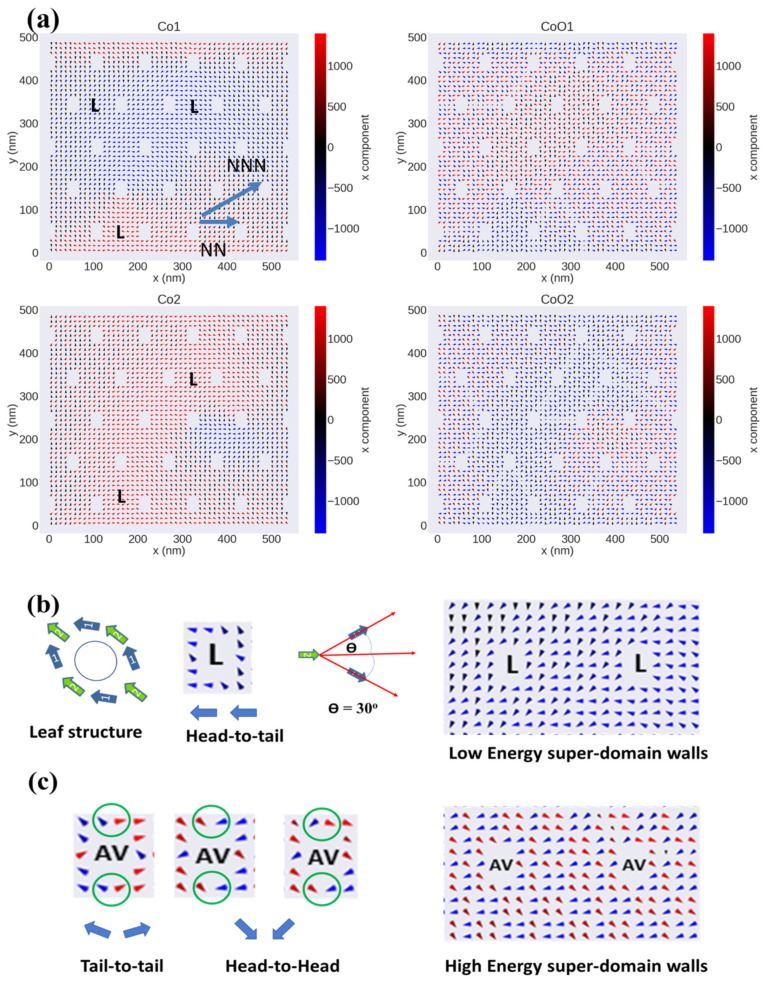
(**a**) The micromagnetic simulation of the INP magnetic domain structure of CoO/Co nanostructured thin films with *W* = 76 nm and fixed lattice parameter *p* = 108 nm at the remanence state for the four layers Co1, CoO1, Co2, and CoO2, respectively. (**b**) Schematic of the leaf spin configuration around a single hole. The connection between two neighboring holes with leaf structure, which induced low-energy superdomain walls, is also indicated. (**c**) Magnetic moment spin configuration around the single hole for the FM/AFM layer that induces high-energy superdomain walls, in which the head-to-tail or tail-to-head rules are applied.

Figure 7a represents the magnetic layers deposited onto the glass used for the micromagnetic simulation. A large area with a multimagnetic domain structure is observed in all four simulated layers (Figure 7b). For FM/AFM ML nanostructured samples with different hole diameters, a noticeable change of the micromagnetic structure can be observed: for nanostructured samples with *W* >> 27 nm, the FM layers exhibit a leaf-structured state (the so-called L-state) [33,60] (see Figure 8a,b), in which the angle between the average magnetization surrounded by four holes forming the hexagonal primitive cell (green arrows in Figure 8b) and the average magnetization of the area between two nearest neighbors (blue arrows) is 30° (see ref. [61] for more details). The magnetic anisotropy for these nanostructured samples follows the near-neighbor rule, which predicts that the difference between the orientation of the near-neighbor easy magnetization axis (NN) and the orientation of the next-near-neighbor hard magnetization axis (NNN) for the hexagonal symmetry is 30° [62]. This magnetic domain configuration is the main factor responsible for the increase of both *H*_C||_ and *H*_EB||_ with *W* reduction [34]. Moreover, the connection between holes with L-state with different direction of the magnetic moment distribution generally generates two kinds of magnetic superdomain walls [55]: (1) low-energy superdomain walls (LE-SDWs), for which tail-to-head or head-to-tail rules are applied, and (2) high-energy superdomain walls (HE-SDWs), for which head-to-head and tail-to-tail rules are applied, and which are the main cause of the perpendicular magnetic signal (PMS) [55]. In this sense, HE-SDWs are present in CoO1 and CoO2 layers because of the strong interaction between FM/AFM. In this case, a new kind of magnetic spin moment arrangement around the hole is created, which we call the anti-vortex (AV)-state, where head-to-head or tail-to-tail rules are applied in one or more parts of the area around the hole (see Figure 8c). Therefore, the main mechanism of the magnetization reversal process for the AFM layers is HE-SDWs rather than LE-SDWs magnetic movement.

**Figure 9:**
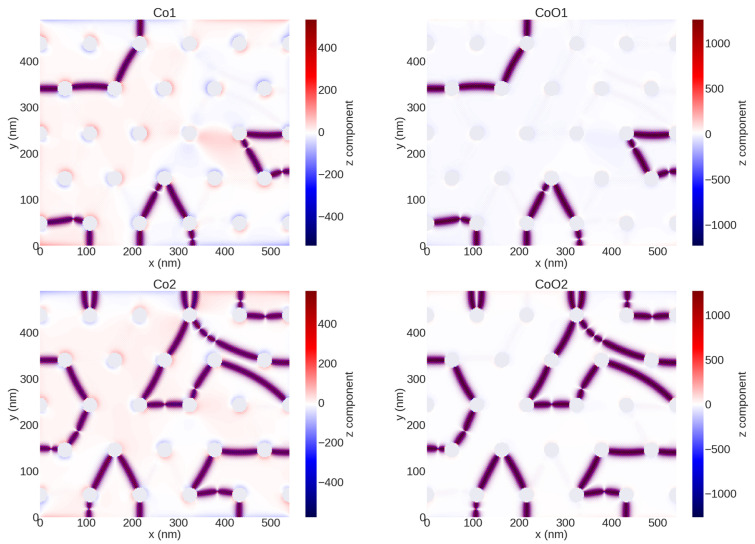
Micromagnetic simulation of the OPP magnetic domain structure of CoO/Co nanostructured thin films with *W* = 76 nm and fixed lattice parameters 108 nm at the remanence state for the four layers Co1, CoO1, Co2, and CoO2, respectively.

By investigating the z component of the magnetization in nanostructured samples with *W* >> 27 nm (see Figure 9), we have found that the Co1 and CoO1 layers show a stripe magnetic domain structure (shown in purple color). We think that this kind of stripe magnetic domain is related to the induced HE-SDWs, as we have explained in the latter paragraph [55]. However, this is not observed in our studies with a lower number of layers (not shown), suggesting that the PMS rises from the formation of AV states as a consequence of the AFM interactions between layers.

**Figure 10:**
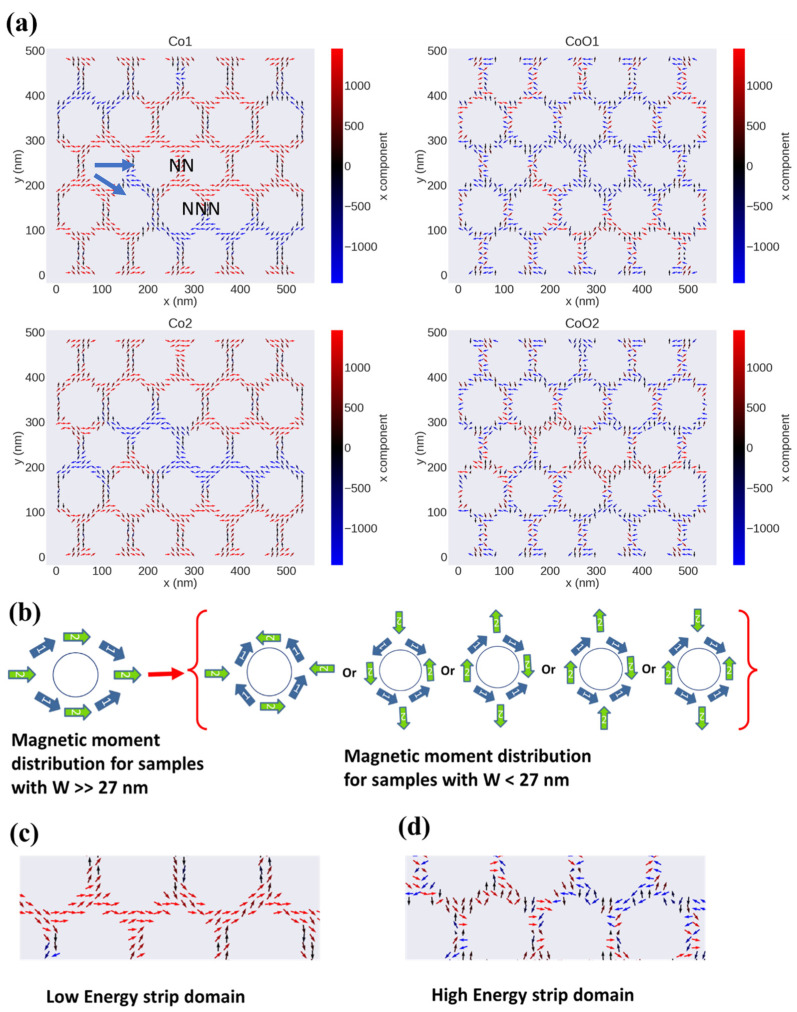
(**a**) Micromagnetic simulation of the INP magnetic domain structure of CoO/Co nanostructured thin films with *W* = 14 nm and fixed lattice parameter *p* = 108 nm at the remanence state for the four layers Co1, CoO1, Co2, and CoO2, respectively. (**b**) Schematics of the spin configurations around the single hole for samples with *W* >> 27 nm and samples with *W* < 27 nm. (**c**) Detail of the low-energy-induced magnetic strip domains for the FM layer, in which the tail-to-head or head-to-tail rules are applied. (**d**) Detail of the high-energy magnetic strip domain for FM/AFM layers where the head-to-tail and tail-to-head rules are applied.

By increasing the hole diameter (i.e., decreasing W below 27 nm), a change in the easy and hard magnetization axes occurs; now the angle between the easy axis and the hard axis is 60° instead of 30°, as sketched in Figure 10 and reported in refs. [61,63]. Therefore, the NNN turns out to be the easy magnetization axis and NN becomes the hard axis of magnetization. This change induces different arrangements of the magnetic moments around the holes that enhance the heat-to-head and tail-to-tail magnetic moment interaction rather than head-to-tail or tail-to-head for the samples with large *W*, as illustrated in Figure 10b. Each head-to-head and tail-to-tail magnetic moment interaction contributes to the appearance of PMS. Moreover, a zigzag structure of stripe magnetic domains is observed, in which the magnetic moment between the holes connects head-to-tail or tail-to-head for the Co1 and Co2 layers. This connection creates a low-energy stripe magnetic domain, as can be seen in Figure 10a,c. Meanwhile, for CoO1 and CoO2 layers, high-energy stripe magnetic domains are created because of the formation of the AV-state. Therefore, the magnetization reversal process for FM/AFM samples with *W* < 27 nm is based on the movement of the low-/high-energy magnetic stripe domain. This scenario agrees with the magnetic domain structure for the z component, in which PMSs are related to the head-to-head or tail-to-tail magnetic moment interaction. The enhancement of PMS for FM nanostructured thin film with *W* < 27 nm was detected and studied in detail in our previous work [31,33,64]. The strong induced PMS in the FM layer is stable enough to overcome the FM/AFM coupling between the layers, as there is no significant change in the PMS for AFM layers.

## 5. Discussion

The main experimental findings of the current investigation concerning these CoO/Co multilayer thin films are related to the observation of a remarkable increase, in more than one order of magnitude, in both the exchange-bias field and the coercivity, when comparing the values of CoO/Co multilayer thin films to those of the non-patterned films. In addition, it is worth noting that the clear dependence of these magnitudes with antidot hole diameters suggested the existence of a crossover between in-plane to out-of-plane magnetic anisotropy at the critical nanohole diameter of ∼75 nm. On the other hand, from the dependence of both coercive and exchange-bias fields with hole diameters, different magnetic arrangements could be suggested in which the magnetization reversal process changes from superdomain wall displacement from a unique low-energy configuration to coherent magnetization rotation. In fact, these experimental results were supported by micromagnetic simulations, in which the existence of antiferromagnetic layers generating an exceptional magnetic configuration around the holes could play a major role in the magnetic process. After considering different magnetic arrangements, we have come to the conclusion that the observed magnetic behavior could be attributed to changes in the superdomain wall pinning in the AFM and in FM layers due to the presence of holes. In this sense, the nanoholes modify the magnetic domain structure, leading to a so-called antivortex state configuration that could be responsible for inducing extra high-energy superdomain walls for samples with edge-to-edge distance *W* >> 27 nm and high-energy stripe magnetic domains for *W* < 27 nm, so that the two anticipated regimes are disclosed. Thus, the present findings suggest a possible mechanism that reinforces the presence of perpendicular magnetic moments in these CoO/Co multilayer thin films with this morphology by tuning the alumina template nanohole size, although it is likely that the direction of the magnetic easy axis is tilted with respect to the sample surface. Further investigations are needed to elucidate this issue.

## 6. Conclusions

We have successfully used a low-cost and effective approach for the synthesis of ultradense CoO/Co sub-30 nm nanoarrays with different geometric parameters. This technique uses conventional nonporous anodic alumina membranes as templates in combination with thermal evaporation deposition of a metallic Co thin film, and enables the precise control of the magnitude of the magnetic anisotropy and the exchange bias of the nanostructured samples. The obtained results propose a unified explanation of the magnetic behavior of AFM/FM antidot array thin films along with the modification of magnetic anisotropy, *H*_C||_ and *H*_EB||_ behavior with antidot hole diameter, and edge-to-edge distance that occurs at the critical antidot geometric points (*W* = 27 nm, *D* = 75 nm). At these critical values of antidots, the maximums of *H*_C||_ (for the entire temperature range) and *H*_EB||_ (at 60 K) are reached, a consequence of the competition between two different complex domain-wall pinning mechanisms occurring among the neighboring holes and the inner wall of the holes. *H*_C||_ and *H*_EB||_ begin to decrease for *D* ˃ 75 nm and *W* < 27 nm due to the misalignment of the AFM and FM spins, the formation of an incomplete AFM domain wall, and the weakening of the AFM anisotropy. The enhancement of the out-of-plane component of the magnetization easy direction was detected for antidot samples with *D* ≥ 85 nm (i.e., *W* ≤ 23 nm) in the temperature range (300 K–150 K). For deep understanding of the magnetization reversal mechanism of the nanostructured samples with different geometric parameters, a micromagnetic simulation by OOMMF was conducted. The strong FM/AFM coupling induced a unique distribution around the hole (called AV-state). These magnetic configurations enhance the PMA signal by creating extra HE-SDWs and HE-strip magnetic domains. As a major outcome, this work provides a successful fabrication route of large areas of multifunctional nanostructures with improved magnetic properties. The remarkable enhancement of perpendicular magnetic anisotropy and exchange bias interactions in these nanostructures can be useful in CoO spintronic applications, such as thermo-magnetic recording patterned media, giant or tunneling applications, and magnetoresistance based on template-assisted deposition methods.

## Figures and Tables

**Table 1:** Geometrical parameters [pore diameter, *D*_p_, and *W*_p_ (= *p* − *D*_p_)] of NAA templates as a function of *t*_etch_. In addition, the magnetic surface coverage ratio percentage, *C*, for [CoO/Co] ML antidot array thin films with different hole diameter, *D*, and separation distance *W* (= *p* − *D*) between holes for the nanopatterned samples. A sketch of the geometrical parameters is shown in Figure 1h.

Sample	Time Etching (min)	*D*_p_ (nm)	*W*_p_ (nm)	*D* (nm)	*W* (nm)	*C* (%)
*S*_TF_	-	-	-	0	-	100
*S*_1_	25	34 ± 3	73 ± 3	32 ± 1	75 ± 2	92
*S*_2_	34	64 ± 3	40 ± 3	61 ± 3	43 ± 4	69
*S*_3_	48	78 ± 2	24 ± 3	75 ± 3	27 ± 3	51
*S*_4_	65	87 ± 4	23 ± 3	85 ± 3	23 ± 2	44
*S*_5_	75	96 ± 3	11 ± 3	94 ± 3	12 ± 2	29

## Data Availability

The data presented in this study are openly available in Materials Cloud at 10.24435/materialscloud:jg-e7.
